# Book Notices, Etc.

**Published:** 1868-12-15

**Authors:** 


### *Book Notices, etc.*

Clinical Lectures on Diseases of the Liver, Jaundice and Abdominal Dropsy. By Charles Murchison, M.D., F.R.S., etc., etc. New York: William Wood & Co., publishers, 61 Walker Street. 1868. Pp. 556.

A compact, perspicuous, and valuable little book, which fills a want long experienced by the profession. We cordially commend it to our readers. Since Dr. Budd's excellent treatise, we have met none on the subjects treated which has given us more pleasure in its perusal.

A large number again crowded out. We trust both authors and readers will have a patience we can not ourselves claim.

